# Are medical schools hesitant to teach undergraduate students teaching skills? A medical student's critical view

**DOI:** 10.3402/meo.v18i0.22997

**Published:** 2013-11-13

**Authors:** Lukas Peter Mileder

**Affiliations:** Clinical Skills Center, Medical University of Graz, Graz, Austria

**Keywords:** medical education, resident-as-teacher training programs, teaching skills, didactics, student-as-teacher training

## Abstract

Junior medical staff provides a large proportion of undergraduate student education. However, despite increasing numbers of resident-as-teacher training programs, junior doctors may still not be sufficiently prepared to teach medical students. Hence, medical schools should consider implementing formal teaching skills training into undergraduate curricula.

Physicians are responsible for patient care, fulfill research duties, and teach students as well as clinical faculty. To meet the requirements of such a demanding and diversified professional field, a wealth of cognitive, psychomotor and behavioral skills, and great enthusiasm for the medical profession are a *conditio sine qua non*. The foundation is laid during medical school, where theoretical and practical aspects of medicine, professional behavior, ethics, and principles of scientific work and research are comprehensively taught. Still, the transition from medical student to graduate doctor is often a challenging process, to say the least. Among the responsibilities of newly graduated doctors, medical practice and teaching engagements are probably the most time-consuming tasks. It has been shown that between one- and two-thirds of student education is provided by junior medical staff (1). However, many residents lack formal training and, therefore, teach students ineffectively (2). As a consequence, resident-as-teacher initiatives have been introduced and described in the medical literature, but it remains elusive if these programs consistently result in improved learner performance (1, 3). But why not address this teaching skills deficit at an earlier stage? The implementation of teaching skills training into undergraduate medical education would seem to be the logical preventive measure, and several authors have stressed this need (4, 5). Still, from both my personal experience and when considering medical education literature, such undergraduate teaching programs are missing or primarily carried out as elective courses by medical schools (6, 7).

Medical education in Graz has a century-long successful history and student peer teachers are widely employed throughout the curriculum, both during the preclinical and clinical periods of study. A myriad of lectures, seminars, and workshops on didactics and professional teaching is offered to postgraduate faculty; yet, to my knowledge there is still not a single student-as-teacher course – neither compulsory nor voluntary – as part of the undergraduate curriculum.

I have been working as a student instructor at our skills laboratory for more than 3 years, teaching fellow students clinical skills and practical procedures from a wide range of specialties. Throughout this period, I have participated in numerous technical skill courses myself but, in contrast, I did not receive any didactic training. All I know about teaching originates from personal experience (‘trial and error’), from observing experienced faculty and fellow student peer teachers performing their craft and, of course, from the medical education literature. It can be argued that teaching instruction may *per se* not be a core task of medical schools, but can and should medical schools rely on undergraduate students’ personal interest and intrinsic motivation to find out themselves how to become qualified clinical teachers of tomorrow? As a matter of fact, the answer has to be an unequivocal ‘no’.

Medical students not only have the desire to assume teaching responsibilities but also are interested in improving teaching skills through formal education before residency (8). The fact that medical practice regulations declare doctors responsible for ‘developing the skills and practices of a competent teacher’ further emphasizes the need for undergraduate teaching skills training (9). A literature review on this topic revealed three major reasons why such programs should be implemented: (i) medical students are future faculty members with teaching responsibilities, (ii) medical students will become more effective communicators, which will improve physician–patient interaction, and (iii) medical students may become better learners through a comprehensive understanding of teaching and learning principles (6).

A number of publications have described successful ‘teaching to teach’ programs (10–12). The study by Zijdenbos et al. (12) is of special interest as it describes mandatory 1-week teaching training for final-year medical students and analyses corresponding course evaluations. Based on 5 years of experience and more than 1,000 graduated students, the authors reported increased interest in teaching among participants and concluded that such programs are a valuable addition to the core medical curriculum. Encouraged by these promising reports, we are currently working on a teaching skills tutorial aiming at our skills laboratory instructors. An elective course open to all interested students is on our agenda and will hopefully be realized within the next two study years.

The implementation of additional courses into an existing curriculum certainly has an impact on other aspects of the program. Curriculum development is a zero-sum game, which means that adding one part to the puzzle will inevitably require removing another. However, curriculum development is also a continuing process of priorities, and medical schools have to consider and evaluate their individual needs carefully. Teaching training for undergraduate students clearly should be among the priorities of medical schools, as the success of every profession depends on future generations being both enthusiastic and competent. Just to wait for excellent medical teachers to step out of the shadow while ignoring the glaring need to actively motivate and train interested prospects could simply be seen as negligent. Therefore, medical schools should actively allocate course time to mandatory training in teaching skills at the undergraduate level. This would be one important step to ensure that medical education of tomorrow will be in good and well-equipped hands.
